# Capsiate ameliorates secondary hyperparathyroidism by improving insulin sensitivity and inhibiting angiogenesis

**DOI:** 10.1111/jcmm.18202

**Published:** 2024-04-09

**Authors:** Peiting Li, Jianda Zhou, Tianyin Wang, Jun Li, Wei Wu

**Affiliations:** ^1^ Department of Plastic Surgery The Third Xiangya Hospital, Central South University Changsha China; ^2^ Transplantation Center The Third Xiangya Hospital, Central South University Changsha China; ^3^ Department of Breast Thyroid Surgery The Third Xiangya Hospital, Central South University Changsha China

**Keywords:** angiogenesis, capsiates, oxidative stress, secondary hyperthyroidism

## Abstract

Secondary hyperparathyroidism has a significant impact on the overall well‐being of the body. Capsiates, known for their antioxidant and metabolic properties, have emerged as a promising alternative treatment for secondary hyperparathyroidism. This study aims to evaluate the effects and mechanisms of capsiates in the treatment of secondary hyperparathyroidism. To achieve our research objectives, we conducted a study on patients' serum and examined changes in metabolic markers using serum metabolomics. We induced secondary hyperparathyroidism in rat through dietary intervention and divided them into four groups. The first group, referred to as the Parathyroid Hormone (PTH) group, received a low‐calcium and high‐phosphate diet (0.2% calcium, 1.2% phosphorus). The second group served as the control group, receiving a standard phosphate and calcium diet (0.6% calcium, 0.6% phosphorus). The third group, called the capsiates group, consisted of rat from the control group treated with capsiates (intraperitoneal injection of 2 mg/kg capsiates for 2 weeks after 2 weeks of dietary intervention). The fourth group was the capsiates‐treated PTH group. Subsequently, we conducted ribose nucleic acid (RNA) sequencing on parathyroid gland cells and evaluated serum thyroxine levels, oxidative stress, expression of proteins associated with vascular neogenesis, measurement of SOD, GSH and 3‐nitrotyrosine, micro‐CT and histological staining. The serum metabolomic data revealed a significant decrease in capsiate levels in the secondary hyperparathyroidism group. Administration of capsiates to PTH rat resulted in increased calcium levels compared to the PTH group. Additionally, the PTH + Capsiates group showed significantly lower levels of PTH and phosphate compared to the PTH group. The PTH group exhibited a notable increase in the quantity and size of mitochondria compared to the control group. Following capsiates administration to the PTH group, there was a significant reduction in the number of mitochondria and length of microvilli, but an increase in the size of mitochondria compared to the PTH group. Sequencing analysis revealed that vascular endothelial growth factor (VEGF) and Vascular Endothelial Growth Factor Receptor 1 (VEGFR1) play crucial roles in this process. Vascular‐related variables and downstream signalling were significantly elevated in hyperthyroidism and were alleviated with capsaicin treatment. Finally, combining capsiates with the PTH group improved bone mineral density, Tb.N, BV.TV, Cs.Th, Tt.Ar, OPG, Ob.TV and Oc.TV, as well as the mineral apposition rate, but significantly decreased Tb.Sp and Receptor Activator for Nuclear Factor‐κ B Ligand (RANKL) compared to the PTH group. The findings suggest that capsiates can improve secondary hyperparathyroidism and ameliorated osteoporosis outcomes by inhibiting angiogenesis and reducing oxidative stress.

## 1 INTRODUCTION

The thyroid gland is a crucial endocrine gland that produces thyroxine, including triiodothyronine and thyroxine, which have significant effects on energy utilisation, protein synthesis and hormone sensitivity.^1^, ^2^, ^3^, ^4^, ^5^ The principal endocrine regulator responsible for calcium levels in humans is parathyroid hormone (PTH).^6^ PTH and PTH‐related peptide (PTHrP) are members of the PTH peptide family, and both are important in maintaining calcium‐phosphate balance and controlling bone metabolism.^7^ Hyperparathyroidism, defined by increased PTH production and high blood PTH levels, can result from parathyroid abnormalities. This can happen either inside or outside the parathyroid gland (primary or secondary).^8^, ^9^ Secondary hyperparathyroidism (SHPT) symptoms include joint discomfort, kidney stones and weakening bones. Secondary hyperparathyroidism is prone to cardiovascular hazards, such as cardiac and cerebrovascular accidents, vascular calcification and other problems^10^; secondly, it can cause severe generalised itching of the skin, and if calcification occurs, the situation may be even more serious, and even skin ulcers and necrosis^11^; and there is also the possibility that it may lead to bone damage, including bone pain, decreased bone density, fibrous osteitis, etc. pathological fractures and bone deformities, which will undoubtedly cause a greater impact on the patient's quality of life and working ability.^12^, ^13^, ^14^ SHPT is complex and varied, seriously jeopardising patients' quality of life. However, there is no standardised treatment approach, especially in terms of surgical intervention and choice of clinical drug therapy.^14^, ^15^ As a result, it is critical to comprehend the effects of SHPT on the body and investigate various treatment possibilities.

Total parathyroidectomy with forearm transplanting has been utilised to treat secondary hyperparathyroidism (SHPT) in patients of severe hyperparathyroidism that does not respond to medication therapy.^16^ Curative parathyroidectomy, on the other hand, might result in chronically increased levels of parathyroid hormone (PTH), delaying post‐operative symptom recovery.^17^ Although auto‐transplantation partially restores PTH secretion, the transplanted tissue's ability to adapt to changes in serum calcium remains significantly impaired.^18^ There are currently no exact strategies for suppressing PTH release in both in situ and transplanted human parathyroid gland (PTG) tissues, particularly those based on blood calcium concentrations.^19^, ^20^

Capsiates are non‐stimulating lipid analogs of capsaicin with antioxidant characteristics and the ability to reduce fat accumulation and induce apoptosis. Capsiates' unique biological properties make them intriguing for treating hyperparathyroidism symptoms. However, there has been little investigation into the effects of capsiates on the parathyroid system. Supplementation with capsiates significantly enhances muscular endurance and improves ischemia–reperfusion injury, obesity and osteoarthritis.^21^, ^22^, ^23^, ^24^

This study aimed to investigate the preventive impact and pathogenesis of capsiates on SHPT by assessing their effects on parathyroid gland cells and hormone levels in both human subjects with SHPT and a mouse model.

## 2 MATERIALS AND METHODS

The research study received approval from the ethical committee of our hospital and the institutional review board (IRB), and all 14 participants provided written consent after being informed about the study. The study adhered to the CONSORT criteria and the Declaration of Helsinki. Animal experiments were conducted in compliance with the ARRIVE guidelines and relevant legislation.

### 2.1 Patients

The study involved 7 healthy volunteers and 7 patients diagnosed with secondary hyperthyroidism, based on iPTH levels greater than 240 pg/mL and serum calcium levels equal to or greater than 8.4 mg/dL. All patients met the following inclusion criteria: (1) confirmed diagnosis of chronic renal failure (uremia stage), (2) no improvement in symptoms after 3–6 months of standard medical therapy, and blood PTH levels greater than 1000 ng/L, (3) severe hypercalcemia or hyperphosphatemia, more than one parathyroid hyperplasia lesion on ultrasonography with a diameter greater than 1 cm and (4) normal liver function and blood coagulation parameters. Exclusion criteria included primary hyperparathyroidism, severe illnesses affecting other organs, inability to cooperate during the study and subsequent follow‐up.

### 2.2 Serum metabolic profiling

Using both targeted and nontargeted metabolomics techniques, the study performed serum metabolic profiling. Targeted metabolomics employed LC–MS/MS to assess capsiate levels, whereas the nontargeted approach used ESI‐QTOF/MS and UPLC‐QTOF/MS. Following data integration, normalization and peak intensity alignment, the positive data set yielded a list of metabolites in each sample, along with corresponding intensities for m/z and retention time. Using the SIMCA‐P software program (v13.0, Umetric, Umea, Sweden), the processed data set was subjected to principal component analysis (PCA) and orthogonal to partial least squares‐discriminate analysis (OPLS‐DA) with a VIP >1 threshold. Thermo Scientific Prelude SPLC was used for chromatographic separation, while the Thermo TSQ Vantage triple quadrupole mass spectrometer was used for detection.^21^

### 2.3 Materials

The phosphate, calcium, PTH and creatinine ELISA kits were provided by Nanjing Jiancheng Bioengineering Institute, while the TUNEL staining kit was provided by Roche Corporation. The thyroid pills were supplied by Laiyang Biochemical Pharmaceutical Factory, while capsiates and other compounds of analytical quality used in the research were provided by Sigma Reagent Company.

### 2.4 Secondary hyperparathyroidism rat model

With a few modest adjustments, the rat model of secondary hyperparathyroidism was created as previously reported.^25^ A total of 100 male Sprague Dawley rats, aged between 8 and 10 weeks, were split into four groups according to the diets they were fed: (1) PTH group on a low calcium and high phosphate diet (0.2% Ca, 1.2% P); (2) control group on a normal phosphate and calcium diet (0.6% Ca, 0.6% P); (3) Capsiates group, which comprised rat from the control group treated with capsiates (injected intraperitoneally with 2 mg/kg Capsiates for 2 weeks after 2 weeks of diet fed treated); (4) Capsiates group, which comprised rats from the PTH group treated with capsiates (intraperitoneally injected with 2 mg/kg capsiates for 2 weeks after 2 weeks).^23^

With the exception of the calcium or phosphate concentrations, which comprised 20% protein and 100 IU/100 g of vitamin D (Beijing Keao Xieli Feed Co. Ltd., Beijing, China), the diets were specially formulated. Prior to lentivirus injection, rats were given a diet rich in phosphate and low in calcium (HPLCa) for 7 days. After that, lentivirus was expressed for 15 days while the HPLCa diet was kept the same. After the rat were fed the HPLCa‐diet for 21 days, either light stimulation or parathyroid tissue isolation was carried out. On the 21st day following the start of the HPLCa‐diet, we switched out the diet for regular chow to see if the diet‐induced rise in PTH was reversible. For 2 weeks, all rats were kept in housing between 22 and 25°C on a 12‐h light/dark cycle, with unlimited access to food and water. Blood samples were obtained from sedated animals (intraperitoneal pentobarbital sodium) by aortic puncture for serum biochemical analyses. HE staining techniques and immunohistochemistry were used to verify parathyroid hyperplasia. Following the administration of Capsiates treatment, the rats were slaughtered, and blood samples were promptly taken. The samples were kept cold, at −80°C, until they could be examined more.

## 3 ELISA

Under ether anaesthesia, blood samples were taken from each animal's hepatic vein just before sacrifice to measure total blood levels of phosphate, calcium, PTH and creatinine levels using ELISA, which were represented as ng/ml, g/l, IU/l and ng/ml, respectively.

### 3.1 The morphology, apoptosis and microstructure of parathyroid gland cells

The rat was sacrificed, and the parathyroid glands were removed. The glands were then maintained for an overnight period in 2.5% neutral‐buffered glutaraldehyde and 10% neutral‐buffered formalin. This allowed us to examine the microstructure, ultrastructure and apoptosis of the parathyroid gland cells. Prior to being embedded in paraffin, the tissues were dehydrated in graded ethanol and fixed in 10% neutral‐buffered formalin. A compound microscope was used to look at tissue histopathology and cell death in slices that were cut to a thickness of 5 m. The slices were then stained with a Tunel staining reagent. Additionally, 2.5% neutral‐buffered glutaraldehyde was applied to the tissues, and an electron microscope was used to evaluate the samples.

### 3.2 Transmission electron microscopy

The parathyroid glands were removed right away, chopped into tiny (1 × 1 mm) pieces, and preserved in 0.1 M phosphate buffer (pH 7.4) containing 2.5% glutaraldehyde and 1% paraformaldehyde for 6–8 h at 4°C. We post‐fixed the tissue samples for 2 h at 4°C in 1% osmium tetroxide in the same buffer after washing them in the buffer. Before infiltrating and embedding the tissues in araldite CY 212, we dried them using ethanol concentrations that increased in size. We used a light microscope to examine thick (1 mm) slices that we had stained with toluidine blue. A Reichert Ultracut E ultratome was used to cut thin slices (60–80 nm), which were then positioned on copper grids, contrasted with uranyl acetate and alkaline lead citrate, and examined using a Morgagni 268D transmission electron microscope (Fei Company, The Netherlands) at an operating voltage of 80 kV.

### 3.3 Measurement of SOD, GSH and 3‐nitrotyrosine

We used the inhibition of nitroblue tetrazolium (NBT) reduction method, as reported by Sun et al.,^26^, ^27^ to measure the activity of CuZn SOD. Utilising a spectrophotometer, the absorbance of the reduction product at 560 nm was determined. The amount of protein that inhibited NBT decrease by 50% was determined to be one unit of SOD. The Beutler et al. technique was used to measure GSH levels. The amount of 3‐nitrotyrosine in plasma was measured using the TCS Biological nitrotyrosine ELISA kit (ZID 7500 A, Buckingham, UK). Repeated dilutions of nitrated BSA, which faced off against immobilized nitrated proteins for the polyclonal anti‐nitrotyrosine antibody, were used to build a standard curve.

### 3.4 Western bloting

Using previously published techniques, a western blot analysis was performed.^23^ Anti‐VEGF and anti‐VEGFR1 (Santa Cruz Biotechnology Inc., Santa Cruz, CA), anti‐p38, Akt, Erk and NOS (Cell Signalling Technology, Beverly, MA, USA), and anti‐b‐actin (Sigma‐Aldrich, St. Louis, MO, USA) were among the primary antibodies that were incubated for a whole night at 48°C. Using ECL Plus (Amersham Biosciences Inc., Buckinghamshire, UK), specific proteins were visualized. Using Quantity One software (Bio‐Rad Laboratories, Hercules, CA), band intensities were evaluated and adjusted to actin or total protein levels.

### 3.5 RNA Sequencing

As directed by the manufacturer, genomic DNA was extracted for RNA sequencing using the Omega Fungal DNA Kit D3390‐02. Using a TBS‐380 fluorometer (Turner BioSystems Inc., Sunnyvale, CA), the pure genomic DNA was quantified. PacBio Sequel Single Molecule Real Time (SMRT) and Illumina sequencing technologies were used to sequence the genome. The complexity of the genome was evaluated using the Illumina data.

### 3.6 Histological staining

The proximal tibia from several groups was extracted, and tissue processing and sectioning were carried out appropriately for histological staining. The bone tissue was decalcified in 4% ethylenediaminetetraacetic acid (EDTA) for 30 days after the tissue samples were fixed in 4% PFA for 48 h at 4°C. After that, the tissue was sectioned at 5 μm thickness and fixed in paraffin. Following tissue slices, HE staining was done, and pictures were captured using a microscope (ECLIPSE 50i, Nikon, Japan).

### 3.7 Micro‐computed tomography (Micro‐CT) analysis

The rat's femora and tibiae were removed and submerged in 4% paraformaldehyde prior to micro‐CT scanning in order to perform micro‐CT analysis. The SkyScan 1076 model was utilised for the scanning, and it was configured with software version 2.6, isotropic voxel 11.53 μm, voltage 48 kV, current 179 μA, and exposure period 1800 ms. The program SkyScan NRecon (version 1.6.8.0, SkyScan) was used to perform three‐dimensional (3D) reconstructions with voxels of 8.66 μm. The datasets were reoriented using DataViewer (version 1.4.4.0, SkyScan), and the morphological parameters were computed using CTAn (version 1.13.2.1, SkyScan). The cortical bone was picked 6.5–7.2 mm proximal from the distal femur development plate, while the trabecular bone was selected between 0.1 and 0.8 mm distal from L4.

The entire trabecular bone's volume (TV, m^3^), bone volume (BV, m^3^), bone fraction (BV/TV, m^3^), and bone mineral density (BMD, g/cm^3^) were all included in the quantitative analysis, which was conducted on all the bone areas within the ROI of the 3D images. The attenuation coefficient of two hydroxyapatite phantoms with defined mineral densities of 0.25 and 0.75 g/cm^3^ was used to calibrate the morphometric parameters.

### 3.8 Statistical analysis

Software called SPSS 19.0 was used to do statistical analysis. For single‐group statistical analysis, the Student's *t*‐test was employed, and the results were reported as mean ± SD. For the single‐factor analysis of variance between different groups, the Duncan procedure was applied. The p‐value of less than 0.05 was considered statistically different.

## 4 RESULTS

### 4.1 Capsiates as a Biomarker for Secondary Hyperparathyroidism Patients

**FIGURE 1:**
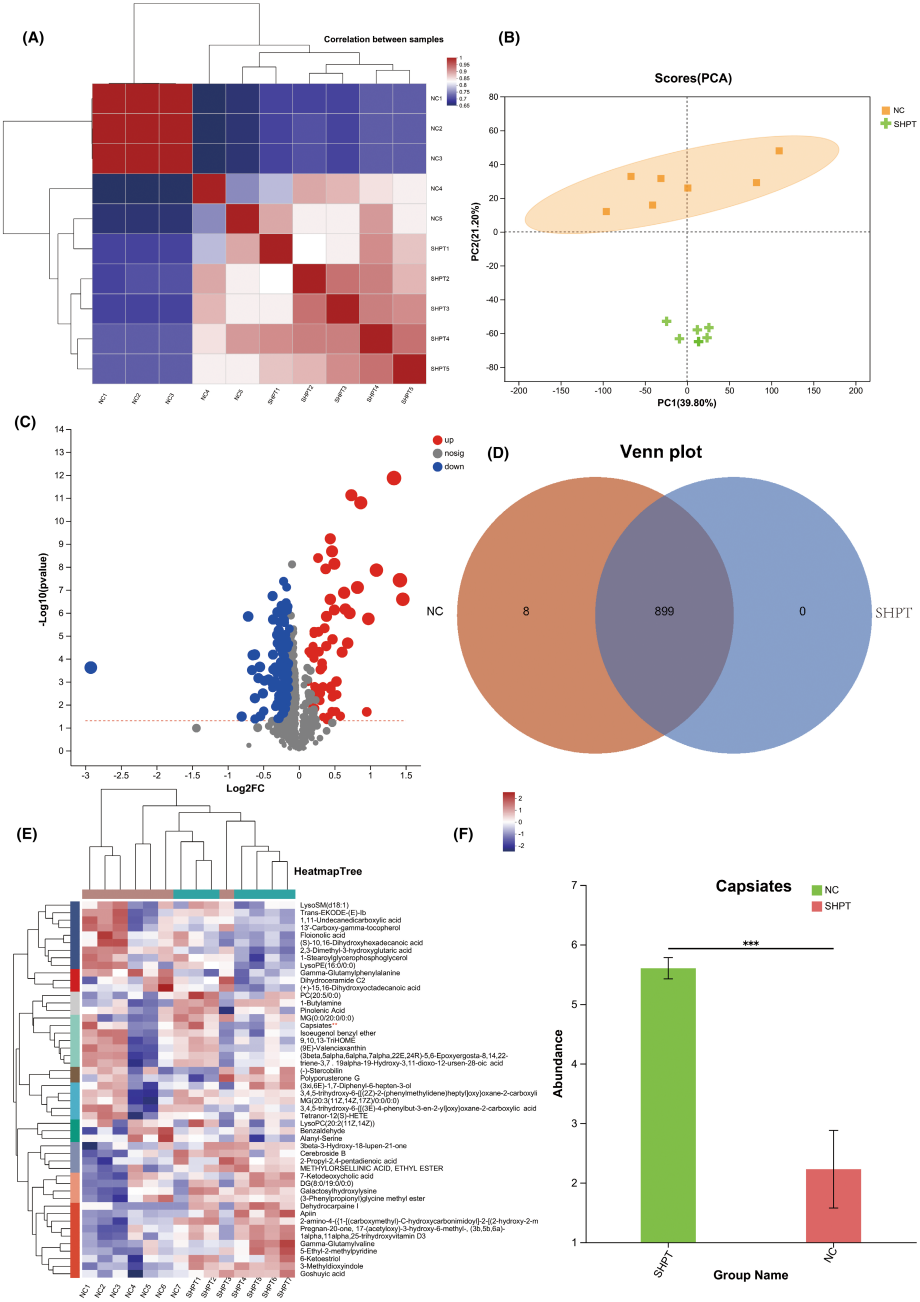
Capsiates as a biological marker in patients with secondary hyperparathyroidism (SHPT). (A) Correlation relationship in patients with SHPT. (B) Principal cause analysis (PCA). (C) Volcano analysis. (D) Venn plot. (E) Heatmap trees. (F) The level of capsiates in patients with SHPT. ***p < 0.001.

The study aimed to evaluate changes in serological metabolites between healthy individuals and those with SHPT. Correlation plots and PCA plots were utilized to assess the distinct difference between metabolites in SHPT and healthy volunteers, as shown in Figure 1. The findings suggest that capsiates are the most prevalent marker of SHPT.

### 4.2 Changes in blood levels of phosphate, calcium, PTH and creatinine

**FIGURE 2:**
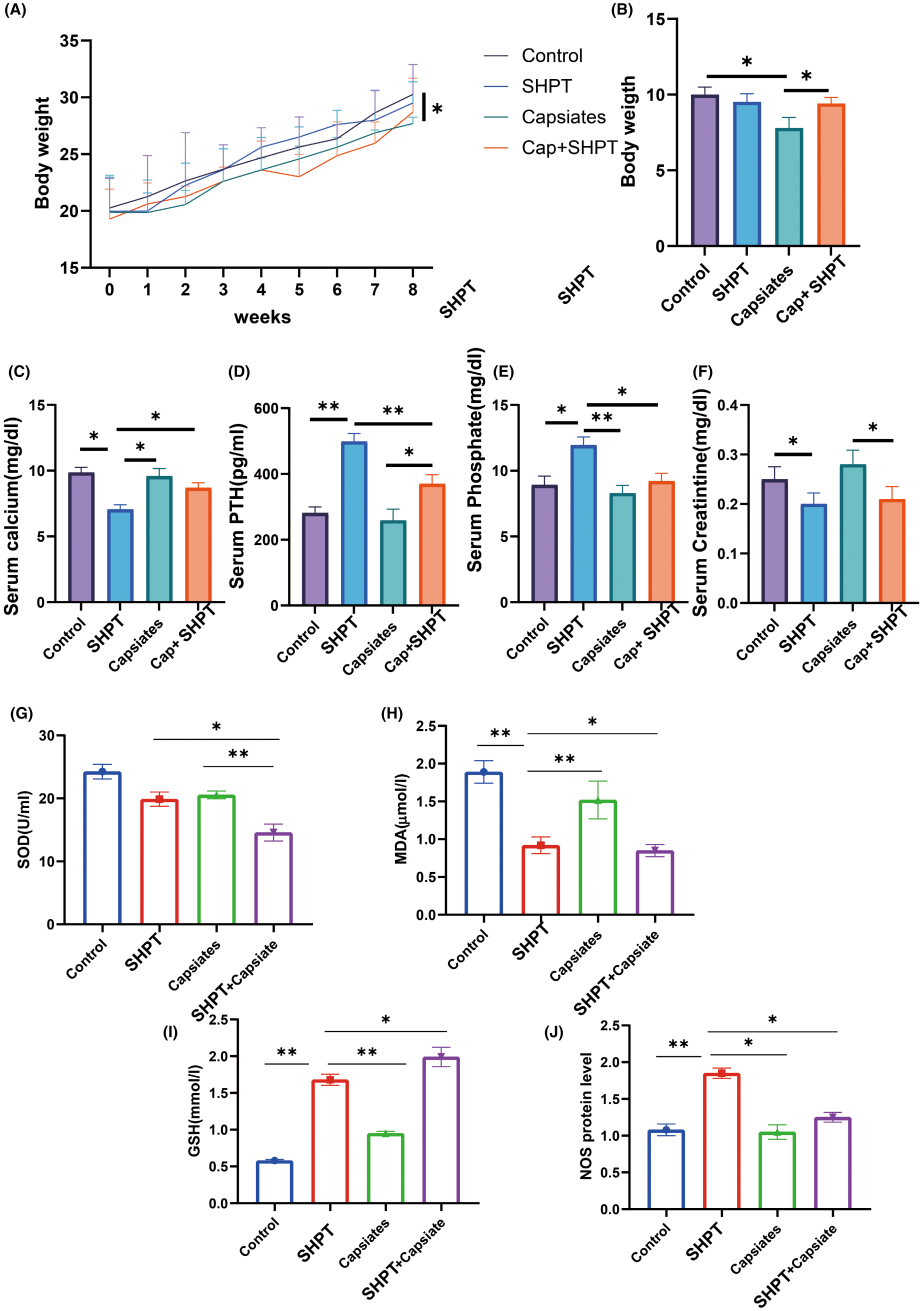
Changes of blood levels of T3, T4, TBG and TSH in hyperthyroidism rat treated with capsiates. (A) Body weight. (B) Body weight gain. (C) Blood levels of T3. (D) Blood levels of T4. (E) Blood levels of TBG. (F) Blood levels of TSH. (G) Epithelial cell height. (H) GSH. (I) MDA. (J) SOD. (K) 3‐nitrotyrosine. *p < 0.05; **p < 0.01. GSH, glutathione; MDA, malonaldehyde; SOD, superoxide dismutase; T3, triiodothyronine; T4, tetraiodothyronine; TBG, thyroxine‐binding globulin; TSH, thyroid stimulating hormone.

Furthermore, changes in blood levels of phosphate, calcium, PTH and creatinine were observed. A low‐calcium and high‐phosphate diet for the SHPT+ capsiates group considerably decreased body weight gain compared to the capsiates group. However, the capsiates group decreased the body weight gain compared to the control group (Figure 2A, B). In addition, the SHPT group significantly decreased the level of calcium and creatinine but increased significantly SHPT and phosphate level (Figure 2C,D). Treatment of SHPT rat with capsiates increased calcium level compared to the PTH group. Moreover, the SHPT+ Capsiates group had significantly lower levels of SHPT and phosphate than the SHPT group, indicating that capsiates exerted a protective effect in secondary hyperthyroidism. These results suggest that capsiates greatly improve hyperthyroidism.

### 4.3 Microstructure and oxidative stress of parathyroid gland cells

The microstructure and oxidative stress of parathyroid gland cells were examined. Our investigation also encompassed the examination of oxidative stress, which revealed significant reductions in MDA levels but increased GSH and NOS levels in the PTH group than control group, while SOD, MDA and NOS levels were significantly decreased in PTH + capsiates than PTH group.

**FIGURE 3:**
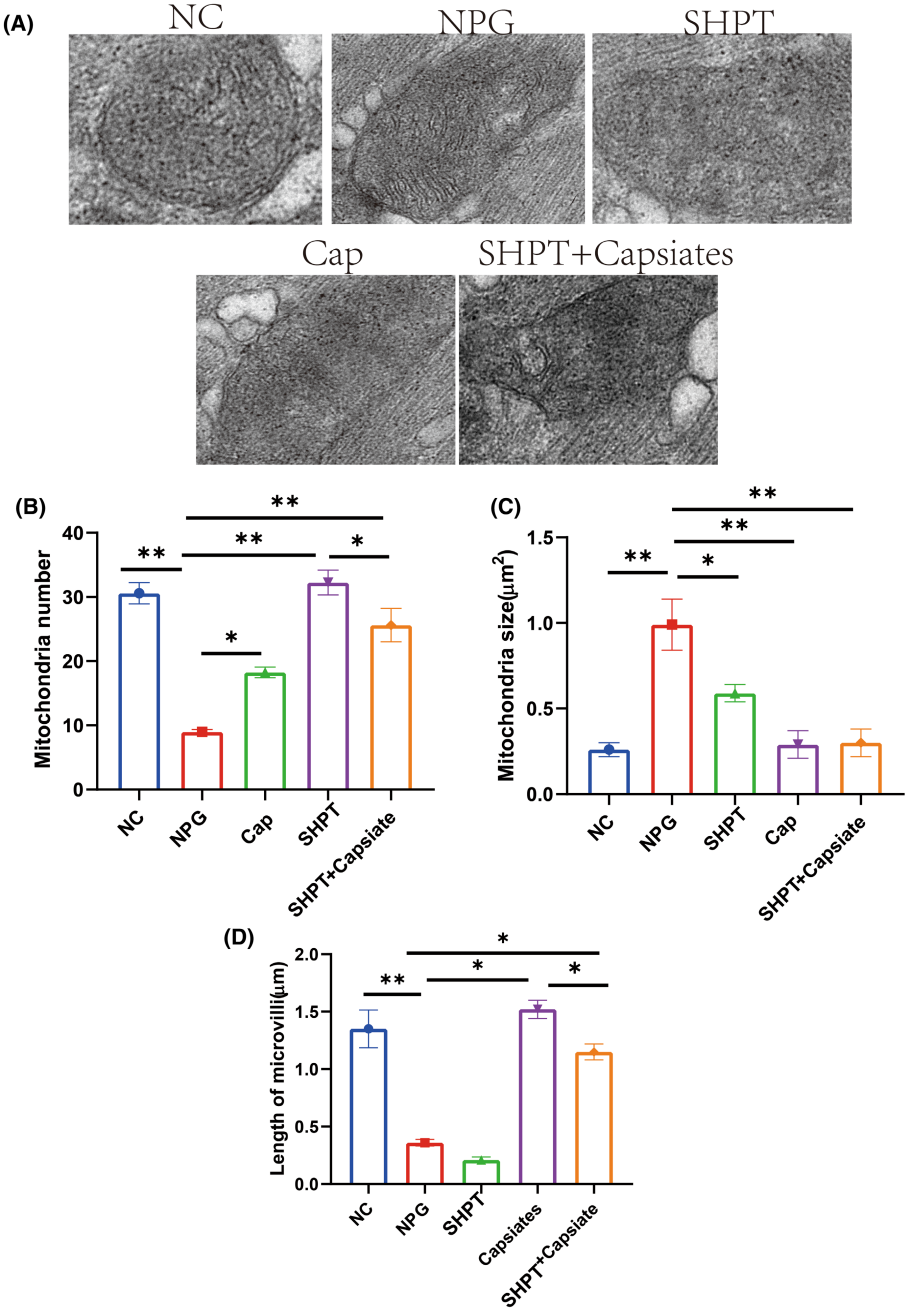
Microstructure and Ultrastructure of parathyroid master cell secondary hyperparathyroidism (SHPT) rat treated with capsiates. (A) Representative mitochondrial diagram. (B) Mitochondria number. (C) Mitochondria size. (D) Length of microvilli. *p < 0.05; **p < 0.01.

Microstructure findings in parathyroid gland cells are shown in Figure 3. There was a significant increase in the number and size of mitochondria in the PTH group than control group. We also observed that following the administration of capsiates group with PTH group had a significantly decreased in mitochondria number and length of microvilli but increased mitochondria size than PTH group. These findings suggest that capsiates have a positive effect on ameliorating oxidative stress levels in parathyroid gland in the secondary hyperthyroidism.

### 4.4 Angiogenesis pathway may play a crucial role in SHPT treated with capsiates

**FIGURE 4:**
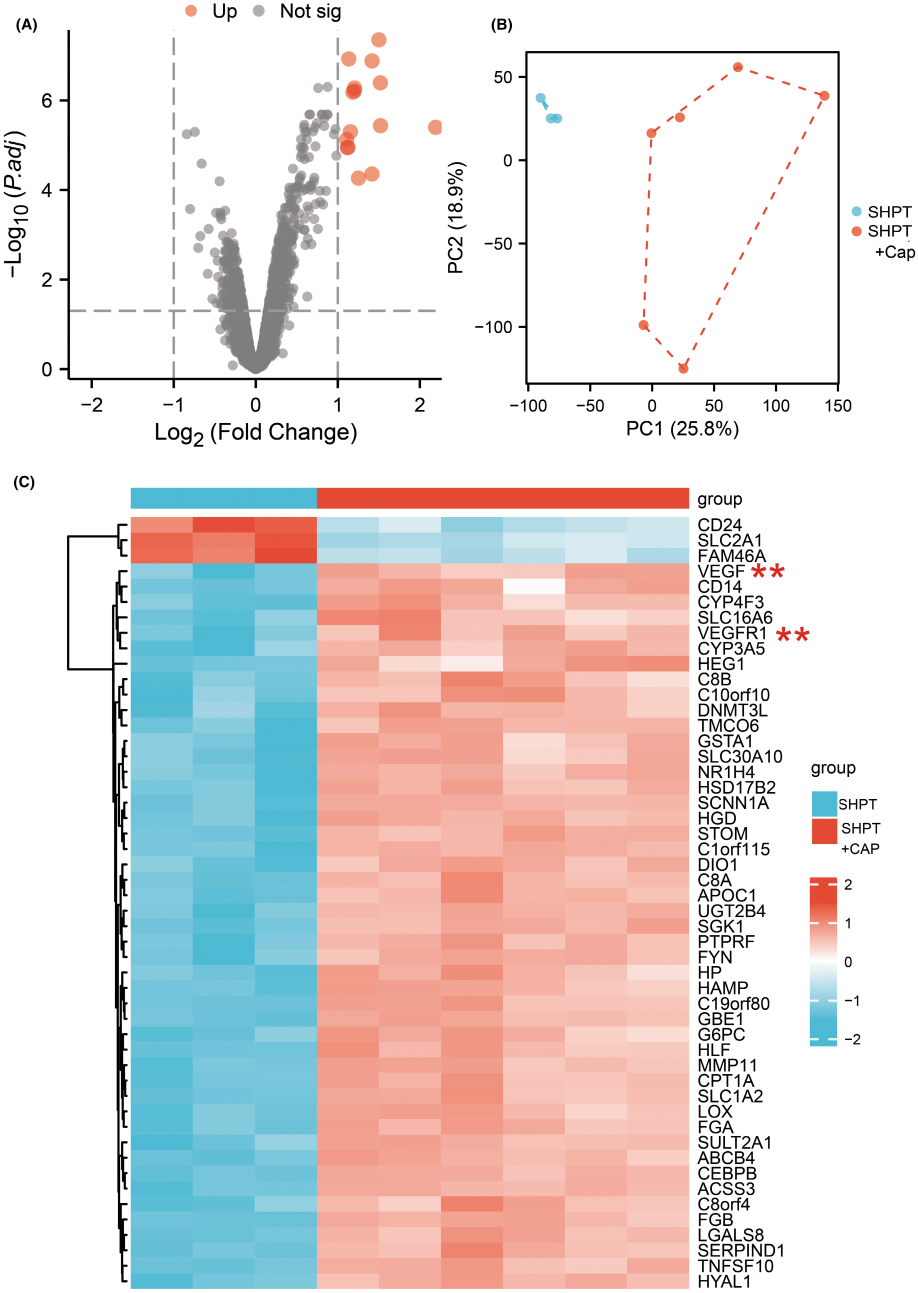
Secondary hyperparathyroidism (SHPT) treated by capsiates were analysed in RNA Sequencing. (A) Volcano analysis. (B) PCA analysed. (C) Heatmap analysis in gene expression. *p < 0.05; **p < 0.01.

**FIGURE 5:**
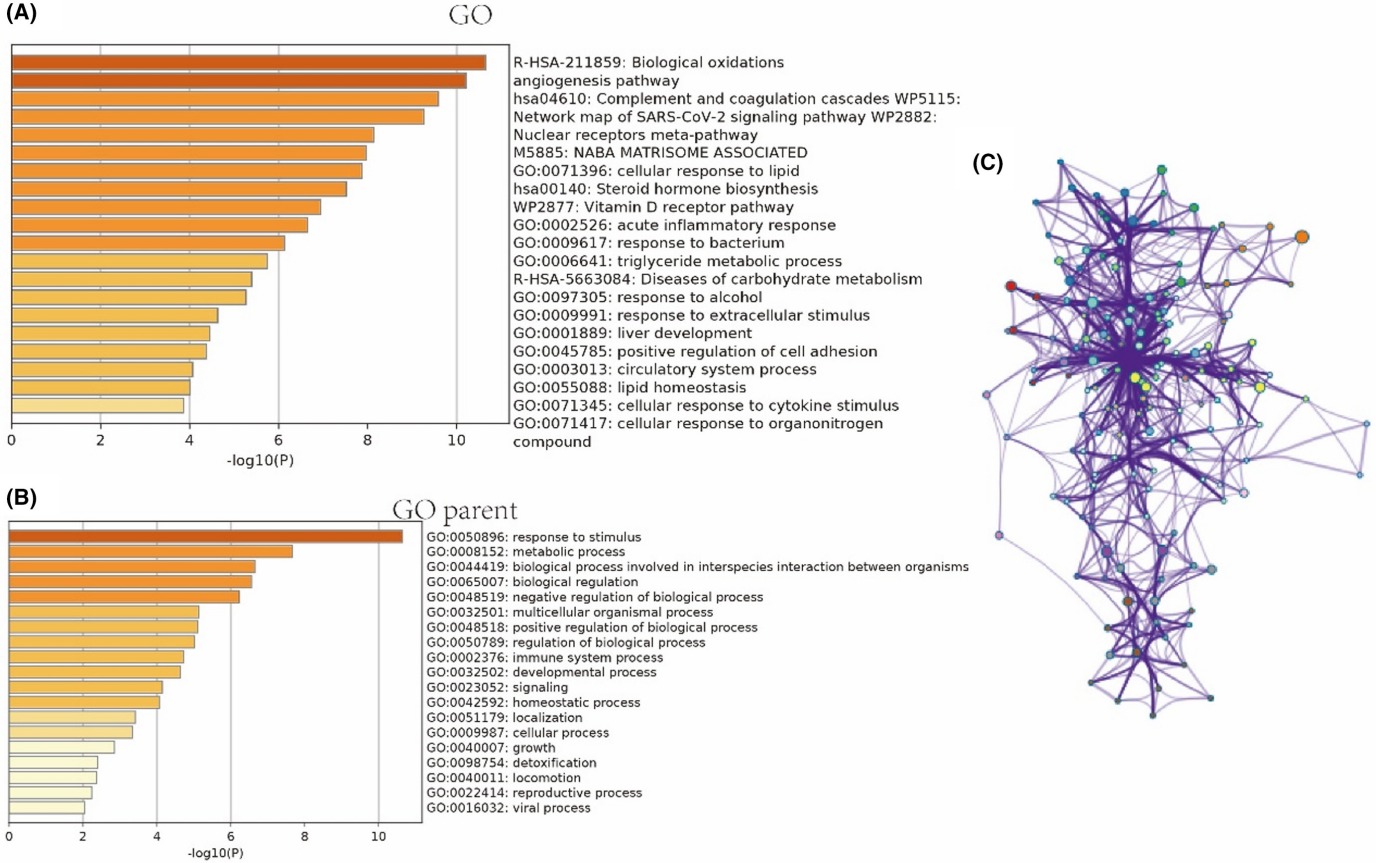
KEGG and GO pathway analysis in secondary hyperparathyroidism (SHPT) treated by capsiates(A–C). KEGG, Kyoto Encyclopedia of Genes and Genomes.

The angiogenesis pathway is believed to play a crucial role in the treatment of SHPT with capsiates. In order to investigate the underlying mechanism of this impact, we conducted a transcriptome sequencing analysis to examine the influence of significant genes and signalling pathways. Our findings revealed that VEGF and VEGFR1 are key factors in this process. Furthermore, functional enrichment analysis of oxidative stress and anti‐angiogenesis signalling pathways suggested that they may also play a vital role (refer to Figures 4 and 5).

**FIGURE 6:**
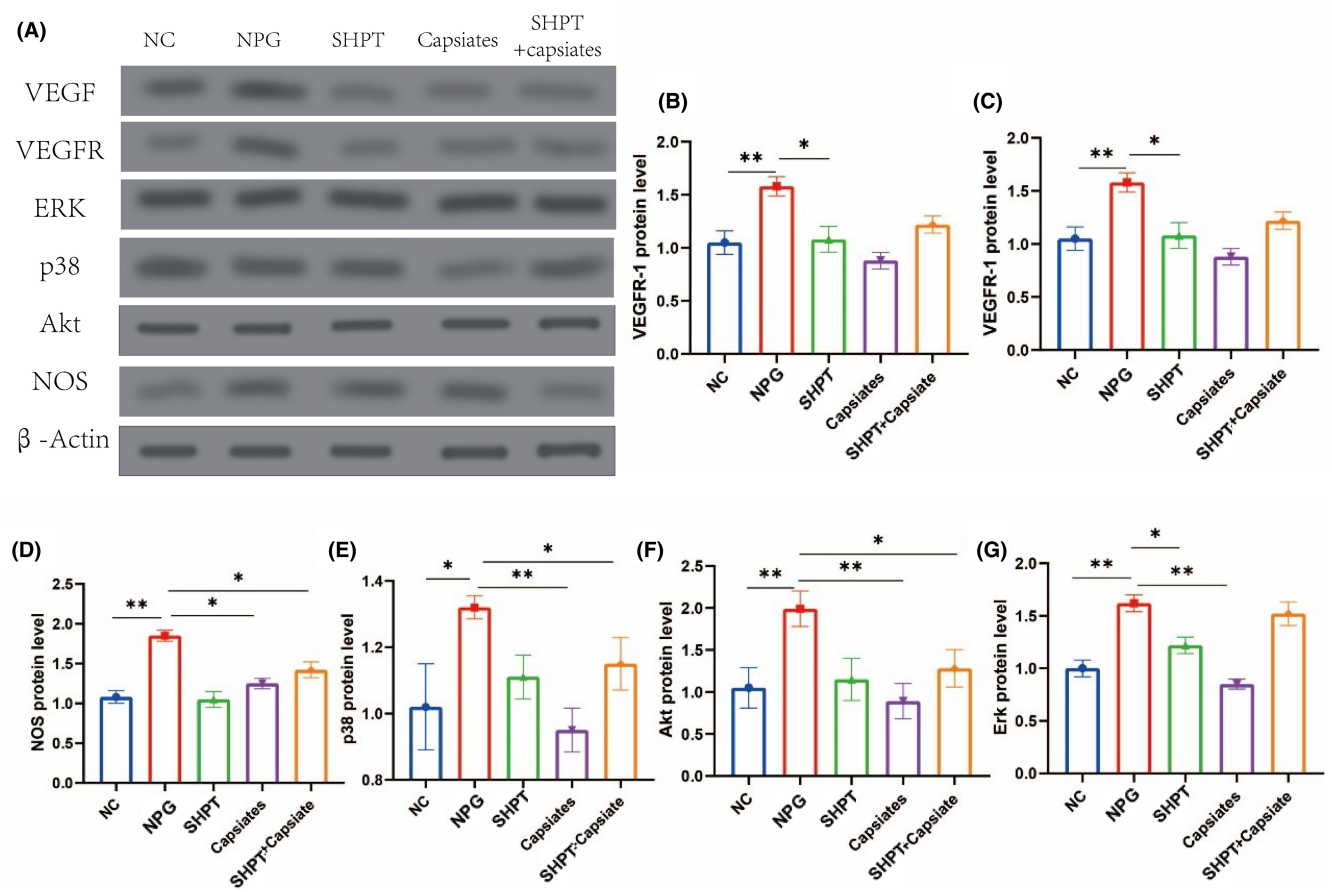
The ERK‐P38 pathway were regulated by VEGF in secondary hyperparathyroidism (SHPT) treated by capsiates. (A) Western blot. (B) VEGF. (C)VEGFR‐1. (D)NOS protein level. (E) p38 protein level. (F) Akt protein level. (G) Erk protein level. *p < 0.05; **p < 0.01. Akt, Serthreonine protein kinase; Erk, Extracellular signal‐regulating protein kinase; NOS, nitricoxidesynthase; VEGF, vascular endothelial growth factor; VEGFR‐1, Vascular endothelial growth factor receptor 1.

Western blot studies demonstrated that SHPT leads to enhanced angiogenesis, including increased production of VEGF and VEGFR1. Additionally, we investigated the downstream signal expression and discovered that Erk, p38, Akt and NOS were significantly upregulated. However, this process was significantly reversed after applying capsiates (refer to Figure 6). Therefore, our study identified possible signalling pathways for hyperthyroidism state with capsiates intervention, which provides evidence for further validation and experimental analysis.

### 4.5 Capsiates intervention reduces SHPT causing osteoporosis

The most prevalent signs of SHPT are decreased bone mineral density and bone fragility. Over time, net bone loss results from persistently high PTH levels in hyperparathyroidism, which also accelerates bone resorption.^28^, ^29^, ^30^ The SHPT group had a substantial rise in Tb.Sp but a significant decrease in bone mineral density, Tb.N, Tb.Th, BV.TV, Cs.Th and Tt.Ar compared to the control group, according to our first analysis of the impact of bone volume and bone microscopic data. Furthermore, compared to the PTH group, capsiates can considerably reduce Tb.Sp while improving Tb.N, BV.TV, Cs.Th and Tt.Ar and bone mineral density.

In addition, we further analysed bone formation and bone destruction indicators. Results of Ob/TV and Mineral apposition rate were lower significantly but Oc/TV have higher level significantly in SHPT group than control group. Capsiates with PTH SHPT group have higher level in Ob/TV and Mineral apposition rate but lower level in Oc/TV than SHPT group.

**FIGURE 7:**
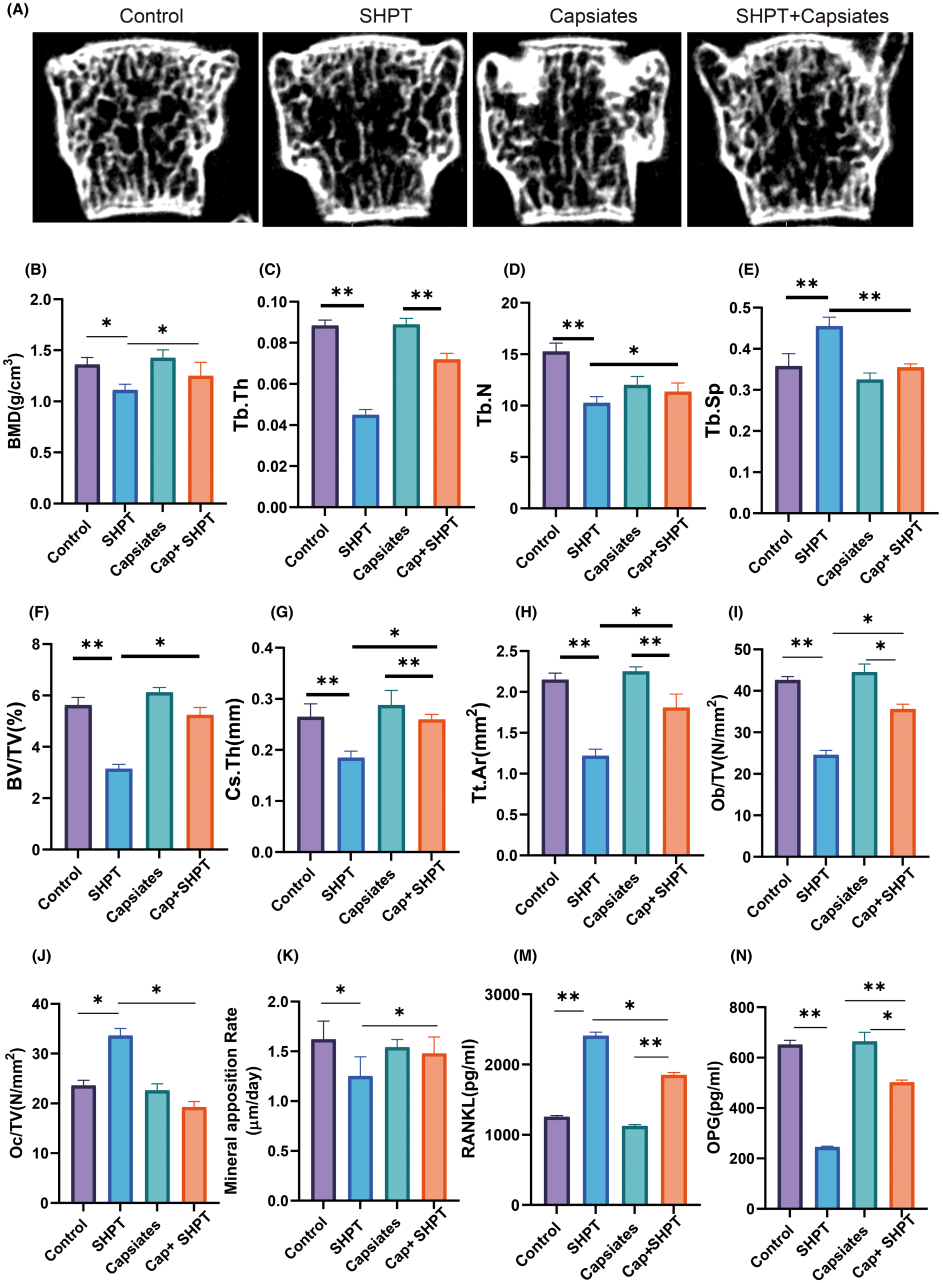
Capsiates intervention reduces secondary hyperparathyroidism (SHPT) causing osteoporosis. (A), Representative graph of Micro‐CT; (B), BMD; (C), Tb.Th; (D), Tb.N; (E), Tb.Sp; (F), BV/TV; (G), Cs.Th; (H), Tt.Ar; (I), Ob/Tv; (J), Oc/Tv; (K), Mineral apposition rate; (M), RANKL; (N),OPG. *p < 0.05; **p < 0.01. BMD, bone mineral density; BV/TV, bone volume fraction; Tb.Th, Bone trabecular thickness; Tb.N, number of bone trabeculae; Tb.Sp, Trabecular separation of bone; Tt.Ar, Total cortical bone area.

Serological results were further analysed RANKL levels were significantly increased in the SHPT group, but capsiates intervention decreased the levels in the SHPT group. OPG level was lower significantly in SHPT group than control group while capsiates with SHPT group increased significantly in OPG level than SHPT group (Figure 7).

## 5 DISCUSSION

Hyperthyroidism is a common clinical condition, especially among older adults, with various risk factors, including women over the age of 60.^31^ Our research found that hyperthyroid patients had significantly reduced serum metabolite capsiates, and animal experiments showed that capsiates supplementation led to significant changes in thyroxine levels and anti‐angiogenic effects in hyperthyroid rats compared to thyroxine tablet supplementation.

Hyperthyroidism affects multiple organs and increases the risk of dyslipidemia,^32^ blood clots and cognitive impairment.^33^ Therefore, it is crucial to initiate therapy and maintain biochemical euthyroidism in hyperthyroid individuals in order to reduce the risk of cardiovascular disease and death.^34^ Additionally, universal screening during pregnancy has revealed that inappropriate secretion of hCG is the most common cause of hyperthyroidism in the first trimester.^35^ Graves' disease, toxic nodular goitre, thyroiditis, medication‐ and iodine‐induced thyroid dysfunction and factitious consumption of excess thyroid hormones are the primary causes of hyperthyroidism.^36^ Over the past few decades, there has been little change in the available therapeutic choices for hyperthyroidism. Although they are a cautious alternative, antithyroid medications (ATDs) have a high recurrence risk. Although radioactive iodine therapy and thyroidectomy are conclusive treatments, they may cause hypothyroidism later on.^37^, ^38^ Thus, the key to a successful course of treatment is comprehending the pathophysiology of hyperthyroidism and determining the best course of action.

One important factor in the pathophysiology of hyperthyroidism is oxidative stress. Given that oxidative stress in humans and animals has been linked to both hyperthyroidism and hypothyroidism,^39^, ^40^ thyroid hormones are especially important in maintaining the antioxidant equilibrium. As shown in vitro and in animals on a diet high in iodide, oxidative stress can also harm the thyroid gland, especially in situations of iodine overload.^41^ Additionally, our study shown that although capsiates can reduce oxidative stress in the PCG and NCG groups, hypothyroidism raises oxidative stress markers in the body. Oxidative damage and the generation of reactive oxygen species (ROS) in the mitochondria are increased in hyperthyroidism.^39^, ^42^, ^43^ Our research also showed that hypothyroidism leads to elevated indicators of oxidative stress in the body, but capsiates can improve oxidative stress in the PCG and NCG groups.

Oxidative damage and the formation of reactive oxygen species (ROS) in the mitochondria are increased in hyperthyroidism.^44^ Rats' hearts experience oxidative stress and altered energy metabolism as a result of thyroid hormone concentrations, which can cause cardiac dysfunction.^45^ According to research by Natalya et al., thyrotoxicosis is characterized by metabolic activation and alterations in the oxidative state. These changes are attributed to abnormalities in the functioning of the respiratory chain and antioxidant enzymes of mitochondria.^46^ Our research confirms these conclusions as we found that supplementing with capsiates alleviated hyperthyroidism‐related increases in mitochondrial number.

Thyroid hormones activate angiogenesis via integrin αvβ3 cell surface receptors. These integrins are made by cancer cells, and they cause blood vessel cells to multiply quickly, which encourages angiogenesis. However, the pro‐angiogenic effect of these integrins can be inhibited by tetraiodothyroglytic acid (tetrac), a deaminated metabolite of T4. While tetrac is not angiogenic, it prevents T4 and T3 from attaching to the αvβ3 hormone receptor region.^47^ This emphasises the necessity of innovative clinical trials and customised theranostic methods since VEGF mRNA isoform specificity in autoimmune thyroid disorders is a major contributing factor.^48^ In thyroid nodules with hyperthyroidism that operate independently, there is a correlation between increased proliferation of hematic vessels but not lymphatic vessels and increased expression of angiogenesis‐related factors.^49^ Clinical studies have shown that angiopoietin‐2 and soluble Tie‐2 could participate in the pathogenesis of Graves' disease and potentially be used as markers of Graves' ophthalmopathy activity.^50^ In our study, we analysed capsaicin's intervention in angiogenesis in detail. Through transcriptome sequencing, we demonstrated that changes in capsaicin levels may be correlated with angiogenesis‐related variables. We also investigated the expression of VEGF and downstream signalling pathway components and found that capsiates were more effective than thyroxine tablets in enhancing thyroid function by decreasing angiogenesis.

In the non‐pungent red pepper cultivar CH‐19 Sweet, the main capsaicinoids are capsiate and its dihydroderivative, dihydrocapsiate.^21^ The link between the vanillyl and acyl moieties is the primary structural difference between capsaicin and capsiate. Whereas capsiate has an ester bond, capsaicin has an amide bond.^51^ Research has also been done on the impact of capsaicin and capsiate on endurance performance.^22^ In the hyperinsulinemic condition, capsiate reduced the generation of glucose by the liver and raised the creation of triglycerides. However, capsiate by itself markedly increased the storage of glycogen, which was linked to increased pAkt→PEPCK and pAMPK signalling.^52^ By upregulating beta‐3‐adrenoceptors in adipose tissue, capsiate may be a viable medication for maximising the anti‐obesity advantages of exercise training, according to Wang et al.^53^ However, capsiate ingestion alone may not be beneficial in treating obesity. The importance of capsiates in thyroid disease is now unclear, nevertheless. Our research showed that, whereas capsiate supplementation considerably reduced the symptoms of hypothyroidism, capsiates were dramatically downregulated in hypothyroidism patients.

Understanding the molecular processes behind capsiate's effects on vascular permeability and angiogenesis has been the focus of recent study. Capsiate has been shown to be effective in reducing VEGF‐induced downstream substrate phosphorylation and Src kinase activation, indicating that it may be used to stop pathological angiogenesis and vascular permeability.^54^, ^55^ Uncertainty surrounds capsiate's possible involvement in oxidative stress. Our research showed that capsiates had a major positive impact on oxidative stress pathways.

Capsiate, a nonpungent analogue of capsaicin, binds to TRP vanilloid 1 (TRPV1) receptor, which is involved in adipogenesis, and could be effective as a bone mineral density agent.^56^ A potential alternative for nonusers of spicy foods who wish to exploit this energy balance property is consumption of nonpungent peppers rich in capsiate, a recently identified nonpungent capsaicin analogue contained in CH‐19 sweet peppers. Capsiate activates transient receptor potential vanilloid subtype 1 (TRPV1) receptors in the gut but not in the oral cavity.^57^ However, the role of capsiate in secondary hyperparathyroidism is currently unknown. In our study, capsiate was found to have a positive effect on improving bone metabolism disorders.

Notably, capsiates may be more effective than thyroxine pills in treating hyperthyroidism. Given the undesirable effects associated with thyroxine pill supplementation, such as cardiovascular events and suppression of the thyroid hormone axis,^58^, ^59^, ^60^ supplementation with capsiates has been shown to have a considerably improved impact on these adverse responses. Therefore, further investigation into the therapeutic efficacy of capsiates for individuals with hyperthyroidism is valuable.

## 6 CONCLUSION

In conclusion, our findings demonstrate a noteworthy reduction in serum metabolic markers, specifically capsiates, among hyperthyroid patients. Furthermore, our results suggest that the amelioration of thyroid‐related hormone levels resulting from capsiates supplementation may be attributed to the enhancement of thyroid epithelial cell morphology and intracellular mitochondrial status, which are closely associated with oxidative stress. Additionally, we observed that capsiates supplementation effectively inhibits angiogenesis of thyroid cells. These findings hold great promise for the development of novel therapeutic interventions for hyperthyroidism.

## AUTHOR CONTRIBUTIONS

**Peiting Li:** Formal analysis (equal); funding acquisition (equal); investigation (equal); methodology (equal); project administration (equal); writing – original draft (equal); writing – review and editing (equal). **Jianda Zhou:** Formal analysis (equal); funding acquisition (equal); supervision (equal); validation (equal). **Tianyin Wang:** Formal analysis (equal); funding acquisition (equal); resources (equal); software (equal); validation (equal); visualization (equal); writing – original draft (equal). **Jun Li:** Conceptualization (equal); data curation (equal); funding acquisition (equal); investigation (equal); supervision (equal); validation (equal). **Wei Wu:** Conceptualization (equal); data curation (equal); investigation (equal); methodology (equal); project administration (equal); software (equal); supervision (equal); validation (equal); writing – original draft (equal); writing – review and editing (equal).

## FUNDING INFORMATION

This research was not funded.

## CONFLICT OF INTEREST STATEMENT

The authors declare no conflict of interest. The funders had no role in the design of the study; in the collection, analyses, or interpretation of data; in the writing of the manuscript, or in the decision to publish the results.

## CONSENT FOR PUBLICATION

We all agree to publication.

## Data Availability

The data used to support the findings of this study are included within the article.
